# Dietary intakes of energy and macronutrients by lactating women of different ethnic groups living in Yakutia

**DOI:** 10.3402/ijch.v72i0.21519

**Published:** 2013-08-05

**Authors:** Tatiana Burtseva, Irina Solodkova, Maya Savvina, Galina Dranaeva, Victor Shadrin, Sergei Avrusin, Elena Sinelnikova, Vyacheslav Chasnyk

**Affiliations:** 1 Yakutsk Research Center for Complex Medical Problems, Siberian Branch of the Russian Academy of Medical Sciences, Yakutsk, Russian Federation; 2 Department of Hospital Pediatrics, Saint-Petersburg State Pediatric Medical University, Saint-Petersburg, Russian Federation

**Keywords:** cross-sectional survey, indigenous people, macronutrients' intake, lactation

## Abstract

**Background:**

There should be a substantial increase in the intake of dietary energy, protein and other nutrients by lactating women, though these special increments can be different in different ethnic groups.

**Objective:**

To evaluate the influence of maternal ethnicity and diet on the quality of breast milk and its potential effect on early childhood development.

**Design:**

A total of 185 mothers (150 Native and 35 Russian) living in settlements and small towns of rural Yakutia and 54 mothers (26 Native and 28 Russian) living in Yakutsk were surveyed and average food intake was recorded during 3 successive days before the survey was analyzed.

**Results:**

The amount of protein varied from 18 to 168.3 g/day, fat – from 12 to 176.1 g/day, energy – from 900 to 3680.4 kcal/day. Protein intake was at the level of current recommended dietary allowances (RDA) in Russians and was higher than in Natives living in rural settlements and small towns (p=0.02) and in Yakutsk (p=0.03). Carbohydrate intake was higher, though not significantly, in both ethnic groups compared with the current recommendations. Protein, fat, carbohydrates and, therefore, energy intake were lower (p<0.03) in Native women living in Yakutsk compared with the intake of Native women living in rural settlements and small towns.

**Conclusions:**

The dietary intakes of energy and macronutrients depended on the place where a woman lived rather than on her ethnicity. Overall, energy intake was considered to be at the lower limit (basal energy expenditure 2002/2005) for lactating women, with the exception of Native women living in Yakutsk whose energy intake was below the lower limit.

It is well known that lactating women should substantially increase their intake of dietary energy, protein and other nutrients. Because breastfed babies of separate ethnic groups living in the same area have different average weights and lengths (1), there is a possibility that the actual intake of energy, protein and other nutrients may differ among different ethnicities.

It is known that, compared with the total population, indigenous people as a rule experience more health-related problems (2).

The results of 2 previous studies performed in 2002–10 were used as a background for this research:

1. Ethnic Native babies were of the same average body weight and length as that of ethnic Yakuts and Russians at birth, although Yakut babies were a bit longer (p<0.04, Table I). The similarities remained regardless of whether or not the family lived in a town or in a small settlement (Table II). At 2–3 years of age, the body weight and stature became ethnicity associated; Native children had shorter stature and lower body weight than Yakut and Russian children (Figs. 1 and 2). The difference in stature and weight between Native children and migrants was so significant that special standards had been issued (5,6) to accurately assess the physical growth and development of Native children.
2. The quality of breast milk (macronutrients) was different in Native women compared with the breast milk of Russian and Yakut women. Milk of Native women was rich in fat and low in protein and carbohydrates compared with milk of Russian and Yakut women living in similar conditions (Table III).

**Fig. 1:**
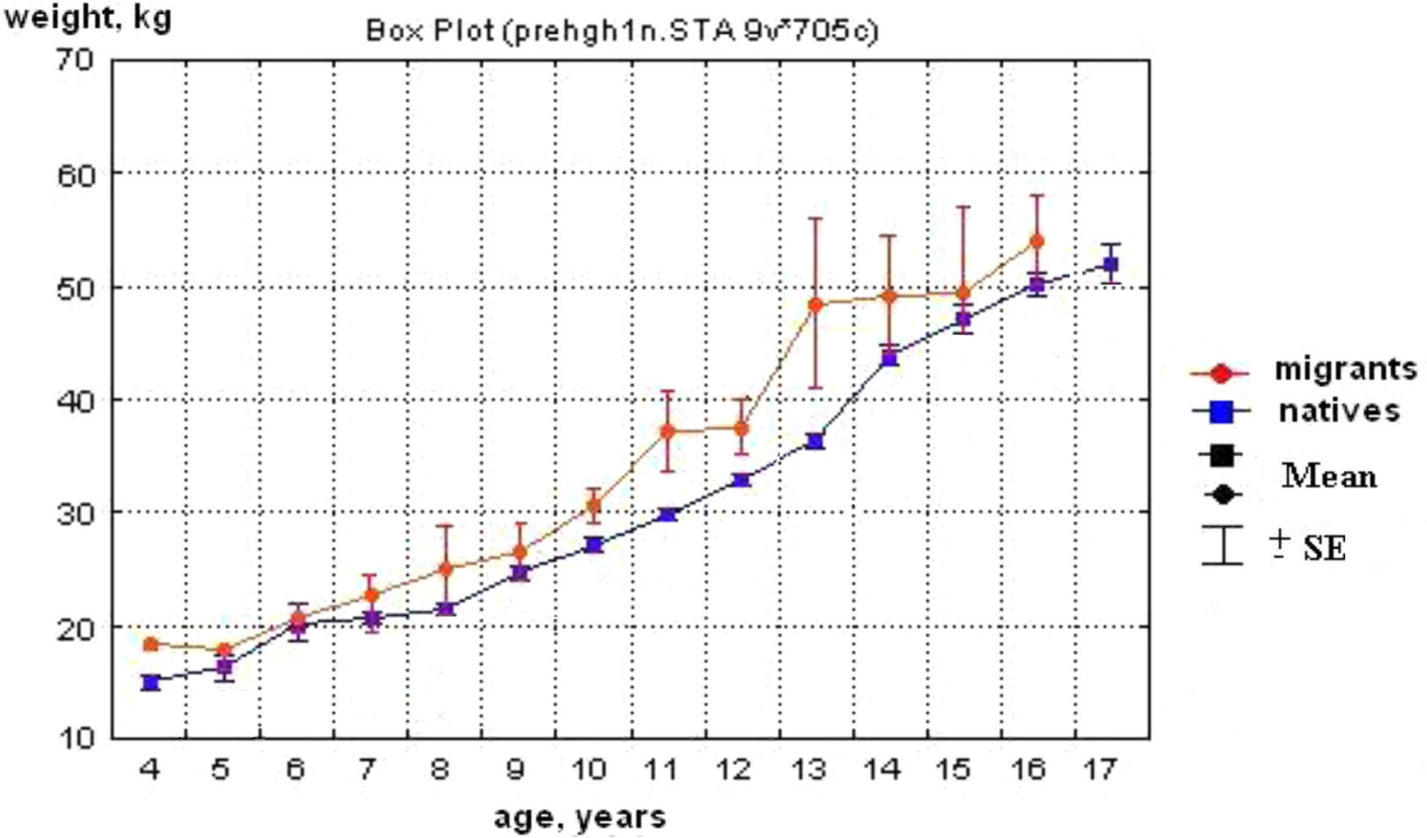
Weight of Native children and of migrants living in the same regions (4).

**Fig. 2:**
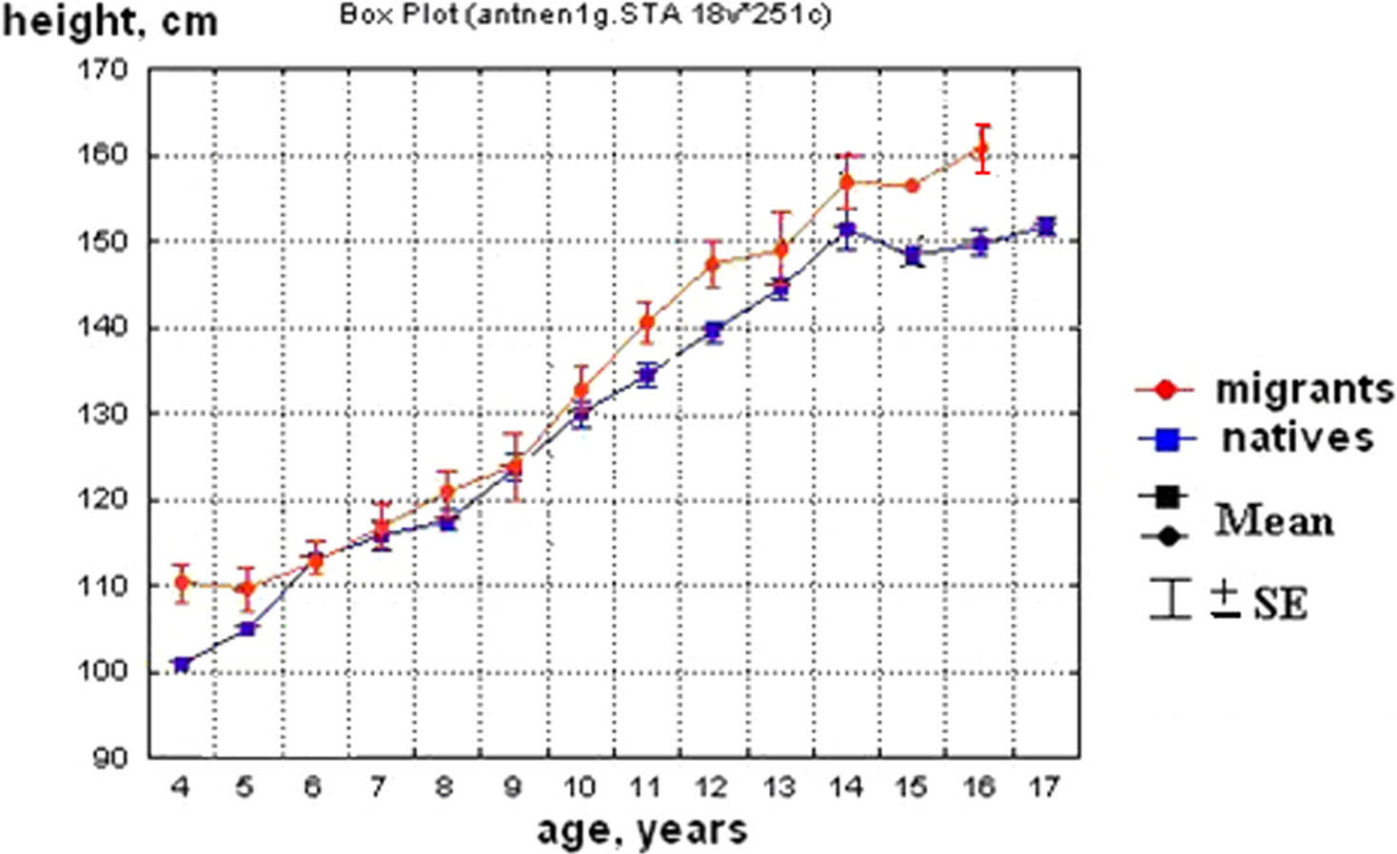
Stature of Native children and migrants living in the same regions (4).

**Table I:** Body length and weight at birth of different Yakutsk ethnic groups (3)

	Body length (cm)		Body weight (g)	
		
Ethnic group	M	S	M	S
Russians	**51.8**	**2.3**	3,409	460
Yakuts	**52.8**	**2.4**	3,511	511
Natives	51.8	3.3	3,356	484

**Table II:** No influence of location on weight and length at birth in different Yakutsk ethnic groups (3)

	Yakuts		Russians	
		
Inhabitation	Average weight (g)	Average length (cm)	Average weight (g)	Average length (cm)
Yakutsk	3,454	52.4	3,457	51.9
Rural areas	3,595	53.5	3,286	51.6
Significance (Student's 2-tailed t-test)	P=0.28	P=0.08	P=0.23	P=0.66

**Table III:** Macronutrients in breast milk of women of different ethnic groups (7)

	Natives vs. Russians	Natives vs. Yakuts	Yakuts vs. Russians
Protein (g/100 ml)	Natives<Russians (P=0.00)	Natives<Yakuts (P=0.00)	Yakuts=Russians (P=0.09)
Lipids (g/100 ml)	Natives>Russians (P=0.00)	Natives>Yakuts (P=0.00)	Yakuts=Russians (P=0.64)
Carbohydrate (g/100 ml)	Natives<Russians (P=0.01)	Natives=Yakuts (P=0.21)	Yakuts=Russians (P=0.11)

## Objective

To evaluate the influence of maternal ethnicity and diet on the quality of breast milk and its potential effect on development in early childhood.

## Materials and methods

**Fig. 3:**
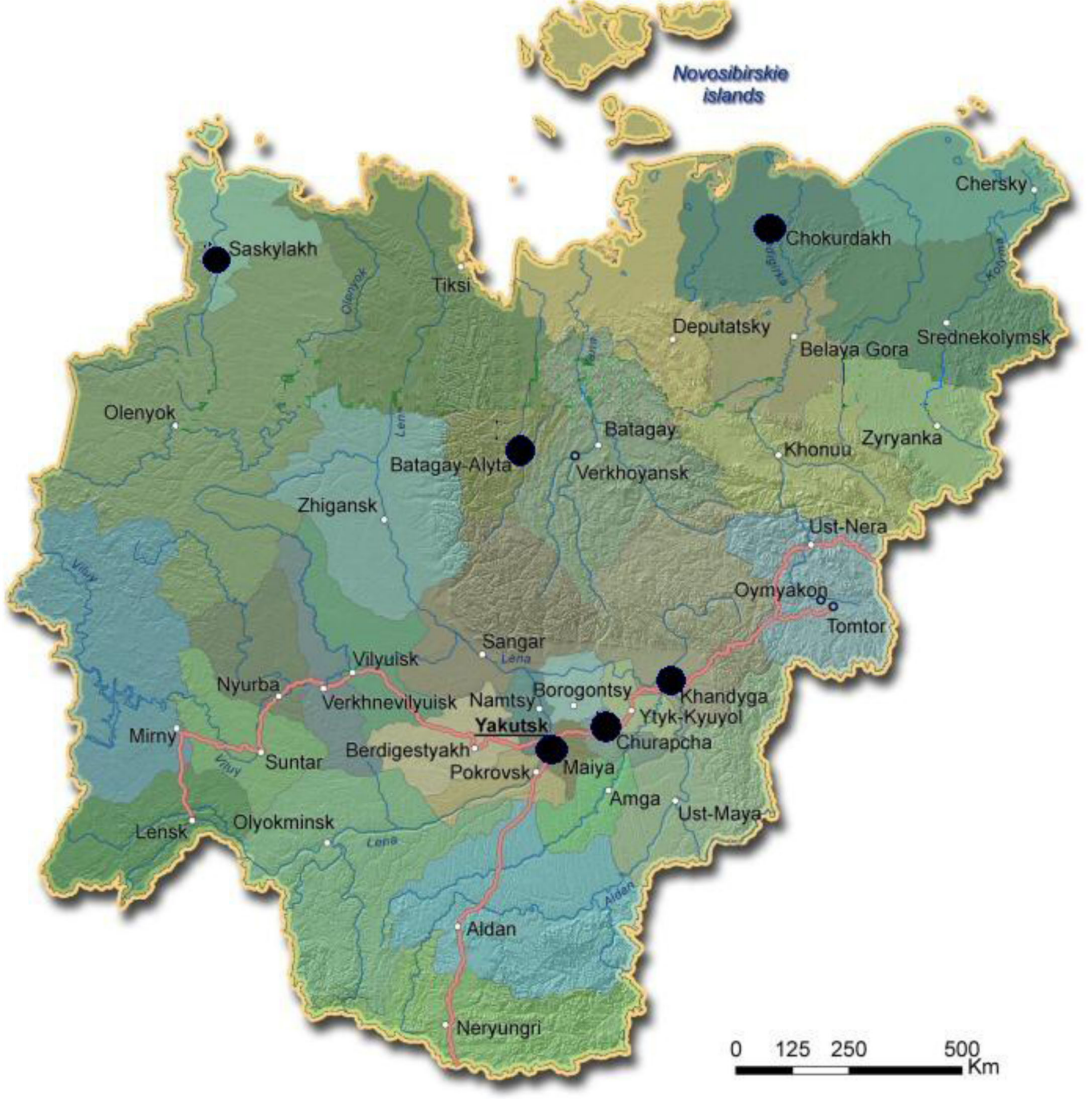
Black dots on the administrative reference map of Yakutia (http://www.yakutiatravel.com/en/map-of-yakutia/adminmap) show the settlements where the survey has been performed.

The study was designed as a cross-sectional personal in-home survey. Of 287 pairs of “mother–baby” living in Yamal and Yakutia that had been studied in the frame of previous research projects, 185 mothers (150 Native and 35 Russian) living in rural settlements in Yakutia (see Fig. 3) and 54 mothers (26 Native and 28 Russian) living in Yakutsk were surveyed.

To be eligible for study enrolment, lactating women had to satisfy all of the following criteria:

1. should reveal their ethnicity
2. should not have any documented chronic disease
3. should be aged between 18 and 45
4. should inform number of previous pregnancies
5. should inform their diet on the 3 successive days prior to the day of survey
6. should provide informed consent signed before the survey.

Informed consent was approved by the Ethics Committee of the Yakutsk Research Center for Complex Medical Problems.

Ethnic group was registered according to self-determination, family history (parents) and phenotype assessed by a local paediatrician (in case of coincidence of these 3 signs).

The lactating women were asked to create a diary of food intake during the past 3 days before the day of collecting the milk sample for analysis and to indicate whether or not it was the traditional menu for the concerned family. For women who informed that it was the traditional menu, the dietary intakes of energy, protein, carbohydrates and fat were calculated using the reference book (8) as an average per day.

Data management and statistical analysis were provided using hard copies of report forms, which were later transferred to electronic copies. We used traditional methods of data management (Microsoft Excel, 73931-640-4347987-57740) and parametric descriptive statistics (Statistica for Windows, ver. 6, AX204B521115F60).

## Results

The amount of macronutrients and energy intake by the women in the study varied and depended on home location, social level and style of living (Tables IV–VI). The amount of protein varied from 18 to 168.3 g/day, fat – from 12 to 176.1 g/day and energy – from 900 to 3,680.4 kcal/day (Table IV).

**Table IV:** Daily macronutrients and calories in diets of lactating women in Yakutia (mean per day for all ethnic groups)

Variable	Mean	S	Max
Protein (g/day)	61.9	25.1	168.3
Fat (g/day)	63.6	33.7	176.1
Carbohydrate (g/day)	253.9	83.6	507.0
Calories (kcal/day)	1819.9	640.4	3680.4

Data presented in Table V show that protein intakes were significantly higher in Russians living in rural settlements and small towns. These intakes were at the level of the current recommended dietary allowances (RDA) for lactating women. Carbohydrate intake was a little bit higher, though not significantly, in both ethnic groups.

**Table V:** Difference in macronutrients and calories intake of Russian and native lactating women living in small settlements

	Mean per day			
			
Variable	Natives, n=150	Russians, n=35	P=(Student's 2-tailed *t*-test)	RDA* (g/day)
Protein (g/day)	**59.8**	**70.7**	**0.02**	71
Fat (g/day)	63.2	65.5	0.72	N/D
Carbohydrates (g/day)	257.6	238.3	0.22	210
Energy (kcal/day)	1819.6	1820.9	0.99	N/D

* Recommended dietary allowances (9).

Data presented in Table VI showed that protein intake was significantly higher in Russians living in Yakutsk.

**Table VI:** Difference in macronutrients and calories in food of Russian and native lactating women living in Yakutsk

	Mean per day			
			
Variable	Natives, n = 26	Russians, n = 28	P=(Student's 2-tailed *t*-test)	RDA* (g/day)
Protein (g)	**53.0**	**69.9**	**0.03**	71
Fat (g)	59.4	68.3	0.31	N/D
Carbohydrates (g)	205.4	233.9	0.27	210
Energy (kcal)	1549.1	1824.6	0.14	N/D

* Recommended dietary allowances (9).

By analyzing data presented in Tables V and VI, we can conclude that protein, fat, carbohydrates and, therefore, energy intake were lower (p<0.03) in Native women living in Yakutsk compared with the intake of Native women living in rural settlements and small towns.

The largest maximum overall daily protein intake was revealed in women living in Yakutsk (168.3 g). This was 20–35% higher than the maximum daily protein intake in small settlements. The largest maximum overall energy intake (2,120 kcal) was observed in women living in the settlement of Saskylakh (Anabar ulus).

## Discussion

There are no evidence-based recommendations existing concerning the intake of dietary macronutrients of a lactating woman, which optimizes the physical development of a baby.

Total energy intake for a lactating woman is calculated at 1.3 times the basal metabolic rate (BMR) or basal energy expenditure (BEE), estimated using the Harris Benedict equation:

BMR(kcal/day)=9.56*weight(kg)+6.25*height(cm)-4.68*age(y)+655(10)

According to current recommendations for BEE, any excess in calories should roughly equal milk energy output (9):

1,200–1,400kcal/day(BEE)+600kcal/day(milk)=1,800–2,000kcal/day

According to both methods, energy intake for a lactating woman should be approximately 1,800–2,000 kcal/day. On average, all participants had an intake almost at the lower limit with the exception of Native women, living in Yakutsk who had an intake below the lower limit and Saskylakh (Anabar ulus), which was the only region where the requirements of energy intake were totally fulfilled (average: 1,932 kcal/day, range: 1,600–2,120 kcal/day).

Surprisingly, the macronutrients’ proportion in Native women was closer to recorded levels in European women than to the inhabitants of other northern regions of the Earth. The amount of protein and fat was lower than in the traditional diet of northern people, and the amount of carbohydrates was much higher. Similar results were observed given the current tendencies in changing dietary habits of northern people (11).

Data presented in Table III showed that the amount of protein and carbohydrates in milk of Native women was lower and the amount of fat was higher than the amount of those macronutrients in the milk of Russian women. These differences were thought to be associated with the differences in diet. However, we had no simple explanation regarding the amount of fat.

## Conclusions

The dietary intakes of energy and macronutrients depended on the place where a woman lived rather than on her ethnicity.

The amount of protein, lipids and carbohydrate intake varied greatly within each ethnic group, and were similar to European than to other Northern peoples’ standards.

The amount of protein in the daily diet of Russian lactating women living in both small settlements and in Yakutsk was within the range of the current RDA.

The amount of protein in the daily diet of Native lactating women living in both small settlements and in Yakutsk was lower than that of the diet of Russian women.

The protein, fat, carbohydrate and, therefore, energy intake are lower in Native women living in Yakutsk compared with the intake of Native women living in rural settlements and small towns.

The amount of protein, fat, carbohydrate and energy intake was the same for Russian lactating women living in small settlements and in Yakutsk.

The results helped to explain lower protein concentration in milk of native women but did not explain the higher amount of fat and lower amount of carbohydrates.

Overall, energy intake was considered to be at the lower limit (BEE 2002/2005) for lactating women, with the exception of Native women living in Yakutsk whose energy intake was actually below the lower limit.

Though the weight and length gain were the same for Native and Russian babies during the first year of life, the subsequent “ethnic” differences can be explained by the differences in diet of lactating women.
